# The chemokine CCL17 is a novel therapeutic target for cardiovascular aging

**DOI:** 10.1038/s41392-023-01363-1

**Published:** 2023-04-19

**Authors:** Yang Zhang, Xiaoqiang Tang, Zeyuan Wang, Lun Wang, Zhangwei Chen, Ju-Ying Qian, Zhuang Tian, Shu-Yang Zhang

**Affiliations:** 1 Department of Cardiology, Peking Union Medical College Hospital, Chinese Academy of Medical Sciences & Peking Union Medical College, Beijing, China; 2 Department of Cardiology, Zhongshan Hospital, Fudan University, Shanghai Institute of Cardiovascular Diseases, Shanghai, 200032 China; 3 Key Laboratory of Birth Defects and Related Diseases of Women and Children of MOE, State Key Laboratory of Biotherapy, West China Second University Hospital, Sichuan University, Chengdu, 610041 China

**Keywords:** Cardiovascular diseases, Translational research

**Dear Editor**,

Aging results in higher susceptibility to age-related disease, especially cardiovascular disease, which has become a public health priority.^1,2^ Recent studies have progressively unraveled the critical role of vasculature as a gatekeeper of life-span and health-span.^3^ In this light, vascular rejuvenation is geroprotective. The circulating proteomic signature is tightly related to aging and aging-induced vascular diseases,^4^ but drugs targeting circulating proteins are not available. Circulating cytokines and chemokines are essential immune components and regulate cardiovascular homeostasis and aging.^5^ Modulating these factors is a potential strategy for treating cardiovascular aging.

We previously demonstrated the involvement of the dendritic cell chemokine CCL17 in coronary artery diseases and cardiac aging.^6,7^ But the pathological functions of CCL17 in vascular aging remain unknown. To investigate the potential pathological roles of CCL17 in vascular biology and diseases, we used a community cohort in the Shunyi district (Beijing, China) enrolling over 1000 participants. We found that serum CCL17 and brachial-ankle pulse wave velocity, a vascular stiffness marker, were increased consistently in the old population compared with young ones (Supplementary Table 1), and CCL17 level was significantly and positively correlated with the stiffness marker brachial-ankle pulse wave velocity (Supplementary Fig. 1). The CCL17 protein level in mouse aortas was also increased by aging or angiotensin II (Ang II) (Fig. 1a), an aging-related vasoconstrictor contributing to aging-related vascular remodeling.^5,8^ These findings suggested CCL17 involvement in vascular aging and dysfunction.

**Fig. 1:**
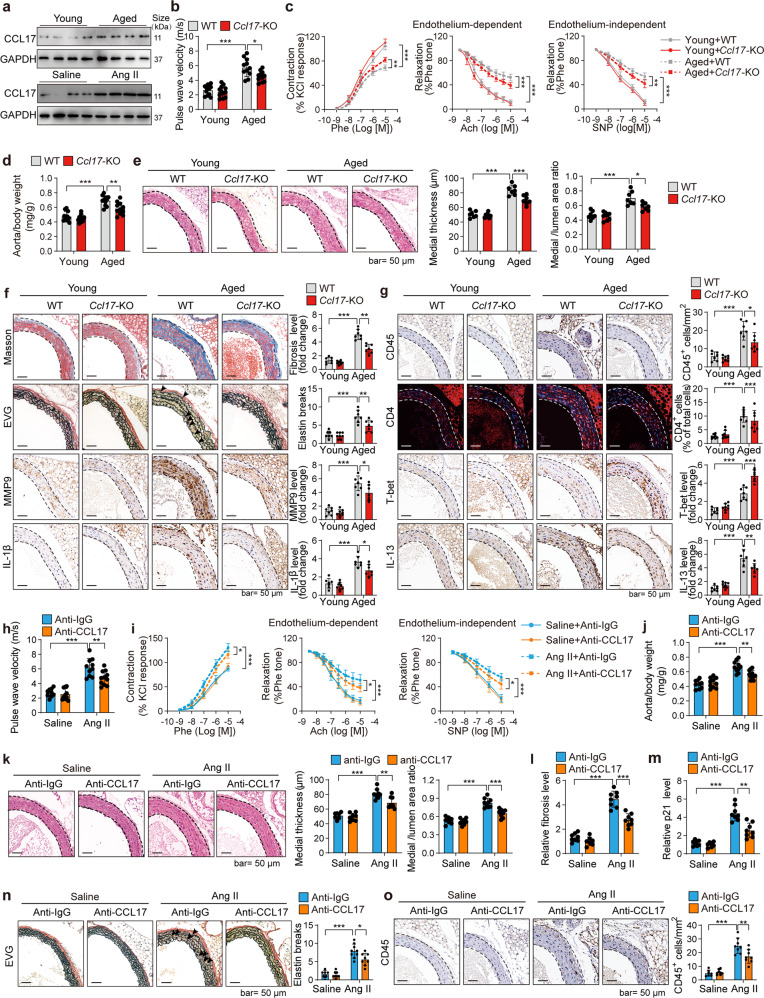
Knockout and inhibition of CCL17 inhibit aging or Ang II-induced vascular dysfunction. **a** CCL17 protein level was increased in the aortas from aged or angiotensin II (Ang II)-treated mice. For the aging model, the aortas of young (4-month) and aged (21-month) mice were analyzed. For Ang II-treated model, Ang II (1.3 mg/kg/day) was applied to challenge young wild-type (WT) C57BL/6 mice for 28 days to induce vascular remodeling, and then the aortas were analyzed. *Ccl17* knockout inhibited aging-induced vascular dysfunction. The aortas of young (4-month) and aged (21-month) WT and *Ccl17* knockout (*Ccl17*-KO) male C57BL/6 mice were analyzed. **b** Pulse wave velocity (PWV) measurement in young and aged mice revealed that *Ccl17* knockout inhibited the aging-induced increase in arterial stiffness (*n* = 11, 12). **c** Ex vivo analysis of the vascular constriction-relaxation function of aortas. (left) the arterial vessel contractions mediated through phenylephrine; (middle) the endothelium-dependent relaxation responding to acetylcholine; (right) the endothelium-independent relaxation responding to sodium nitroprusside (*n* = 6). **d** *Ccl17* knockout reduced the aorta-weight to the body-weight ratio in aged mice (*n* = 11, 12). **e** *Ccl17* knockout reduced vascular remodeling in aged mice; hematoxylin-eosin (H&E) staining of the thoracic aortas was performed, and the quantitative results of the medial thickness as well as the media-area/vessel-lumen ratio are shown (*n* = 7, 8). **f** *Ccl17* knockout reduced fibrosis (Masson staining), elastin fiber breakage (EVG staining), and expression of biomarkers of senescence-associated secretory phenotypes (immunohistochemical staining of MMP9 and IL-1β) in aged aortas. Representative images and quantitative results are shown (*n* = 7, 8). **g** *Ccl17* knockout regulated Th1 (T-bet) and Th2 (IL-13) cells in aged aortas. Immunohistochemical staining was performed to analyze total immune cells (CD45), Th1 marker (T-bet), and Th2 marker (IL-13). Immunofluorescence staining was performed to analyze total CD4^+^ T cells. Representative images and quantitative results are shown (*n* = 7, 8). CCL17 neutralizing antibody inhibited Ang II-induced vascular dysfunction. Young (4-month) WT mice were challenged by Ang II (1.3 mg/kg/day) and treated with CCL17 neutralizing antibody (anti-CCL17, 100 μg per day) or control isotype antibody (anti-IgG, 100 μg per day) for four weeks. **h** PWV measurement revealed that the CCL17 antibody repressed Ang II-induced increase in arterial stiffness (*n* = 9–11). **i** Ex vivo analysis of the vascular constriction-relaxation function of aortas from mice. (left) the arterial vessel contractions mediated through phenylephrine; (middle) the endothelium-dependent relaxation responding to acetylcholine; (right) the endothelium-independent relaxation responding to sodium nitroprusside (*n* = 6). **j** CCL17 antibody decreased the aorta-weight/body-weight ratio in Ang II-treated mice (*n* = 9–11). **k** CCL17 antibody reduced remodeling of aortas in Ang II-challenged mice; H&E staining of the thoracic aortas was performed, and the quantitative results of medial thickness and the media-area/vessel-lumen ratio are shown (*n* = 7–9). **l** CCL17 antibody reduced vascular fibrosis (Masson staining) in Ang II-treated mice (*n* = 7, 8). **m** CCL17 antibody reduced the senescence marker p21 in aortas from Ang II-challenged mice (*n* = 7, 8). **n** EVG staining showing CCL17 antibody reduced breakage of elastin fiber in the aortas from Ang II-challenged mice (*n* = 7, 9). **o** CCL17 antibody reduced immune cell (CD45) infiltration in the aortas from Ang II-challenged mice (*n* = 7, 8). CCL17, C-C motif chemokine ligand 17; EVG, elastic van Gieson; IL-1β, Interleukin-1β; MMP9, matrix metalloproteinase-9. All the data are presented as mean ± SD. When the assumptions were satisfied, a two-way ANOVA test was applied with Bonferroni *post-hoc* test for comparison. Otherwise, we used Kruskal–Wallis test with Dunn’s *post hoc* test for comparison. *P < 0.05, **P < 0.01, ***P < 0.001

In comparison with the increased vascular stiffness in the aged wild-type (WT) mice, the vascular stiffness declined in aortas of the aged mice with *Ccl17* knockout (Fig. 1b). Moreover, *Ccl17* knockout improved aging-induced decline in phenylephrine-induced vascular constriction and endothelium-dependent/-independent relaxation (Fig. 1c), suggesting CCL17 involvement in aging-induced vascular remodeling. Compared with the young WT mice, the aged WT mice had an increased aorta weight to body weight ratio, which was reduced by *Ccl17* knockout (Fig. 1d). The medial thickness and the media-area/vessel-lumen ratio of the thoracic and abdominal aortas were elevated in the aortas of aged WT mice, while this pathological vascular remodeling was also attenuated by *Ccl17* knockout (Fig. 1e, Supplementary Fig. 2). Additionally, *Ccl17* knockout reduced aging-induced vascular fibrosis, breakage of elastin fibers, expression of senescence marker p21 and biomarkers of senescence-associated secretory phenotype (SASP),^8^ including matrix metalloproteinase-2 (MMP2), MMP9, interleukin-1β, as well as monocyte chemoattractant protein-1 (Fig. 1f, Supplementary Fig. 3 and 4). *Ccl17* knockout also alleviated Ang II-induced vascular arterial stiffness, constriction-relaxation dysfunction, pathological vascular remodeling, fibrosis, and senescence-associated secretory phenotype (Supplementary Fig. 5 and 6). Collectively, CCL17 deficiency exerts geroprotective effects on aged arteries.

We next explored how CCL17 regulated vascular aging, thus the expression pattern of CCL17 and its receptor CC chemokine receptor 4 (CCR4) in mouse aortas was analyzed. *Ccl17* was predominantly expressed in dendritic cells, while *Ccr4* was mainly expressed by T cells (Supplementary Fig. 7), suggesting that CCL17 may regulate T cells to regulate vascular aging. Our previous study demonstrated the involvement of helper T (Th) cells in CCL17 function during cardiac aging.^6^ Indeed, *Ccl17* knockout reduced aging-induced infiltration of total immune cells and CD4^+^ T cells in aged aortas. Notably, aging-induced expression of Th1 markers (T-bet, IL-2) as well as Th2 markers (IL-4, IL-13) in aortas, while *Ccl17* knockout increased the overexpression of Th1 markers but reduced Th2 markers in aged aortas (Fig. 1g, Supplementary Fig. 8a). These effects of CCL17 on Th cells were also observed in aortas of Ang II-challenged mice (Supplementary Fig. 8b-d). *Ccl17* knockout also reduced the infiltration of dendritic cells, neutrophils, and macrophages (Supplementary Fig. 8e), which was consistent with our findings in aged hearts.^6^ Thus, CCL17 may regulate Th cells to reprogram the immune microenvironment and act as the orchestrator of vascular aging.

Finally, to test whether CCL17 can be targeted for inhibiting cardiovascular aging, the CCL17-neutralizing antibody was applied to treat Ang II-challenged mice. The serum CCL17 level was increased by Ang II infusion but diminished by CCL17-neutralizing antibodies without any effects on blood pressure or heart rate (Supplementary Tables 2 and 3).^6^ CCL17 antibody repressed Ang II-induced vascular arterial stiffness and constriction-relaxation dysfunction (Fig. 1h, i), and reduced aortic remodeling, fibrosis, cell senescence, and senescence-associated secretory phenotype, elastin fiber breakage, coupled with reprogrammed immune microenvironment (Fig. 1j–o, Supplementary Fig. 9).

The vascular system is the key determinant of aging and lifespan. Vascular rejuvenation can induce healthy aging and extend lifespan in animals.^3^ Here we demonstrated that CCL17 is a key regulator of vascular aging and CCL17 could serve as a therapeutic target. Previous works from our laboratory and other scientists have demonstrated that CCL17 regulated T cells to promote cardiac aging,^6,9^ which may be also the potential mechanism underlying vascular aging because we observed such reprogramming of the immune microenvironment (T cells balance) in vascular aging. Further in-depth analysis is still needed to explore CCL17 in reprogramming the immune microenvironment during cardiovascular aging. CCL17 antibody showed therapeutic values in Ang II-induced vasculature dysfunction and remodeling in young mice, further study in aged mice and large animals can provide more evidence for targeting CCL17 in aging treatment. Another limitation is that we used specific pathogen-free (SPF) animals, which differ from ‘normal or wild’ ones in many aspects such as growth rate, social interactions, immune function, and stress response. For instance, SPF rats display abnormal hematological, hemodynamic, and hemostatic phenotypes in response to anesthetics.^10^ This may be a common limitation of most studies on aging and immunology and may complexify the translation of findings to human vascular function. Finally, although our cross-sectional population study revealed CCL17 level was positively correlated with vascular stiffness, future prospective cohort studies exploring the correlation of CCL17 and vascular function in humans would provide more convincing evidence for the intervention of vasculature aging and related cardiovascular diseases in human patients.

Overall, this study was the first to provide evidence that the chemokine CC17 acted as a novel precise target for preventing vascular dysfunction induced by aging or other risk factors such as Ang II or hypertension. Targeting CCL17 is a novel and promising anti-inflammation approach for inhibiting cardiovascular aging. Our study may provide a paradigm targeting circulating proteome for delaying/inhibiting cardiovascular aging.

## Supplementary information

SUPPLEMENTAL MATERIAL

## Data Availability

The data supporting the conclusion of this study can be required from the corresponding authors.

## References

[1] Zhu X (2021). Inflammation, epigenetics, and metabolism converge to cell senescence and ageing: the regulation and intervention. Signal Transduct. Target Ther..

[2] Bloom SI, Islam MT, Lesniewski LA, Donato AJ (2023). Mechanisms and consequences of endothelial cell senescence. Nat. Rev. Cardiol..

[3] Grunewald M (2021). Counteracting age-related VEGF signaling insufficiency promotes healthy aging and extends life span. Science.

[4] Lehallier B (2019). Undulating changes in human plasma proteome profiles across the lifespan. Nat. Med..

[5] Kong P (2022). Inflammation and atherosclerosis: signaling pathways and therapeutic intervention. Signal Transduct. Target Ther..

[6] Zhang Y (2022). CCL17 acts as a novel therapeutic target in pathological cardiac hypertrophy and heart failure. J. Exp. Med..

[7] Ye Y (2015). Serum chemokine CCL17/thymus activation and regulated chemokine is correlated with coronary artery diseases. Atherosclerosis.

[8] Mehdizadeh M, Aguilar M, Thorin E, Ferbeyre G, Nattel S (2022). The role of cellular senescence in cardiac disease: basic biology and clinical relevance. Nat. Rev. Cardiol..

[9] Feng G (2022). CCL17 aggravates myocardial injury by suppressing recruitment of regulatory T cells. Circulation.

[10] Letson HL, Morris J, Biros E, Dobson GP (2019). Conventional and specific-pathogen-free rats respond differently to anesthesia and surgical trauma. Sci. Rep..

