# Supplementary material for: The chemokine CCL17 is a novel therapeutic target for cardiovascular aging
Source: Signal Transduct Target Ther. 2023 Apr 19;8:157. doi: 10.1038/s41392-023-01363-1 (PMC10113193; doi:10.1038/s41392-023-01363-1)
Supplement: Supplementary file 1 — SUPPLEMENTAL MATERIAL [file 41392_2023_1363_MOESM1_ESM.docx]

Supplementary Materials for

**The Chemokine CCL17 is a Novel Therapeutic Target for Cardiovascular Aging**

Yang Zhang^1,2,#^, Xiaoqiang Tang^3,#^ Zeyuan Wang^1,#^, Lun Wang^1^, Zhangwei Chen^2^, Ju-Ying Qian^2^, Zhuang Tian^1,^*, ,Shu-Yang Zhang^1,^*

^1^Department of Cardiology, Peking Union Medical College Hospital, Chinese Academy of Medical Sciences & Peking Union Medical College, Beijing, China; ^2^Department of Cardiology, Zhongshan Hospital, Fudan University, Shanghai Institute of Cardiovascular Diseases, Shanghai, 200032, China; ^3^Key Laboratory of Birth Defects and Related Diseases of Women and Children of MOE, State Key Laboratory of Biotherapy, West China Second University Hospital, Sichuan University, Chengdu 610041, China.

^#^Yang Zhang, Xiaoqiang Tang, and Zeyuan Wang contributed equally to this work

Correspondence to:

Shu-Yang Zhang ([shuyangzhang103@nrdrs.org](mailto:shuyangzhang103@nrdrs.org)) or Zhuang Tian (tianzhuangcn@sina.com)

**This PDF file includes:**

Materials and Methods

Figures. S1 to S9

Tables S1 to S3

Materials and Methods

**Population study**

In this clinical study, 1,041 participants from three natural villages were recruited at Shunyi (Beijing, China) between June 2013 and April 2016.[^1^](#_ENREF_1) All the clinical parameters of the participants are shown in Supplementary Table 1. Every participant signed informed consent.

**Animals and experiment design**

The global *Ccl17* knockout (*Ccl17*-KO) mice (C57BL/6 background) were used as we previously described.[^1^](#_ENREF_1) The mice were kept in condition of specific pathogen-free (SPF), and the male mice were fed for 21 months to study cardiovascular aging.

For the angiotensin II-induced model of cardiovascular remodeling, young male *Ccl17* knockout (*Ccl17*-KO) mice and wild-type littermate mice were subcutaneously challenged by angiotensin II (Sigma-Aldrich, A9525; 1.3 mg/kg per day) for 28 days with the minipumps as we reported previously.[^1^](#_ENREF_1)^,^[^2^](#_ENREF_2) For neutralizing antibody treatment, anti-CCL17 antibodies (R&D Systems, MAB529) and the isotype-control antibodies were injected intraperitoneally (100 μg per mouse daily) as described previously.[^1^](#_ENREF_1)^,^[^3^](#_ENREF_3)^,^[^4^](#_ENREF_4) At the endpoint of the experiments, the blood pressure and heart rate were analyzed. Then, heart tissues and vascular tissues were obtained at the same time, thus the CCL17 serum level and basal information of heart rate and blood pressure are also shown in our previous work.[^1^](#_ENREF_1)

**CCL17 serum level measurement**

The serum levels of the cytokine CCL17 in human participants and mice were monitored using CCL17 ELISA kit (R&D Systems; #PDDN00, #MCC170) as we reported previously.^1^

**Measurement of pulse wave velocity (PWV)**

PWV measurement was performed using a protocol described previously.[^5^](#_ENREF_5) Briefly, the Pulse wave Doppler ultrasound velocity measurement (Visual Sonics Inc., Toronto, ON, Canada) was carried out at distal/proximal location by recording electrocardiogram signals simultaneously for short periods while the mice had stable heart rate as well as breathing rate. We then evaluated the transit-time by minusing of distal arrival time between electrocardiogram R-wave peak and velocity upstroke foot from a similarly determined proximal arrival time. Furthermore, distance between measurement sites was calculated from B-mode images encompassing both distal/proximal location. The proximal velocity measurement was obtained 1 mm downstream of aortic arch. Besides, the brachial-ankle pulse wave velocity (baPWV) in human participants was also measured.[^6-8^](#_ENREF_6)

**Functional analysis**

The aortic rings were isolated from mice, and then the rings were placed on organ baths with 5 mL Krebs solution (4.7 mM KCl, 119 mM NaCl, 2.5 mM CaCl_2_, 25 mM NaHCO_3_, 1mM MgCl_2_, 1.2 mM KH_2_PO_4_, and 11 mM D-glucose). Next, Multiwire Myograph System was used to perform vasoconstriction experiment. Briefly, through lumen, the isolated aortic rings were placed between two stainless steel-hooks. Then an isometric force transducer was applied to monitor the contractile force. The aortic ring was firstly contracted in 60 mM KCl solution and then rinsed with Krebs solution 3 times for equilibration for 45 min. We have adjusted the resting tension to 3 mN. The concentration-response curves of cumulative phenylephrine, acetylcholine, and sodium nitroprusside were finally obtained.

**Histopathological analysis**

For immunohistochemical analysis, mouse aorta tissues were fixed using paraformaldehyde (4%). Then, the fixed aortas were embedded in the paraffin and the aortic tissues were cut to sections, which were then analyzed using a hematoxylin-eosin (H&E) kit (Servicebio, G1005) and elastic van Gieson (EVG) staining (Servicebio, GP1035). For Masson staining to evaluate vascular fibrosis, the Masson staining kit (Servicebio, G1006) was used. The antibodies used for experiments were anti-CD45 (Servicebio, GB113886), anti-p21 (Servicebio, GB11153), anti-MCP-1 (Servicebio, GB11199), anti-MMP2 (Servicebio, GB11130), anti-MMP9 (Servicebio, GB11132), anti-Collagen I (Servicebio, GB11022), anti-Collagen III (Servicebio, GB111629), anti-T-bet (Abcam, GB300451), anti-IL-1β (Servicebio, GB11113), anti-IL-2 (Servicebio, GB11114), anti-IL-4 (Abcam, sc-53084), anti-IL-13 (Solarbio, K003206P). All of the pictures were taken with the same settings.

For immunofluorescence staining, aorta tissues from mice were first fixed in paraformaldehyde (4%) for 24 hours, then the aorta tissues were embedded using OCT. Next, the sections (7 μm) were made and then blocked in 10% bovine serum for 1 hour. The tissues were then treated using antibodies at 4°C overnight, followed by treatment with fluorescent-coupled secondary antibodies for 45 mins and DAPI for 10 min. The primary antibodies anti-CD4 (Servicebio, GB11064), anti-CD11c (Servicebio, GB11059), anti-Ly6G (Servicebio, GB11229), anti-F4/80 (Servicebio, GB113373) were used. The secondary antibodies information (Servicebio, GB25303).

**Western blotting analysis**

The western blotting experiment was used to analyze CCL17 protein levels in aortic tissues. Aortic tissues were subjected to extraction for total proteins with a lysis buffer (Beyotime, P0013B). A standard western blotting experiment was carried out.[^2^](#_ENREF_2) The SDS-PAGE gel was applied to separate total proteins and the proteins were then transferred to PVDF membranes. Next, 5% fat-free milk (TBST) was applied to block the non-specific antigens. Then, the membranes were washed and incubated with individual antibodies at 4°C overnight. The anti-CCL17 antibody was obtained from Abcam (ab182793) and anti-GAPDH from HuaBio (EM1101). HRP-conjugated secondary antibodies obtained from Thermo (31430 and 31460) were applied to incubate the washed membranes. Finally, the protein expression was analyzed by exposing the membranes to Western Blotting Substrate (Thermo, 32106).

**Analysis of CCL17 and its receptor CCR4 in mouse aortas.**

*Ccl17* and *Ccr4* mRNA in mouse aortas were analyzed using the web tool singlecell.broadinstitute.org. The dataset SCP1361 was used as input.[^9^](#_ENREF_9) This data has been deposited into the Single Cell Portal.

**Statistical analysis**

In this study, all the data are shown as mean ± SD. Normality was evaluated by the Shapiro-Wilk or Kolmogorov–Smirnov test. Besides, the variance homogeneity of data was assessed using the Brown–Forsythe test. We used a two-way ANOVA with the Bonferroni *post-hoc* test, when the assumptions were satisfied, for multiple comparison. Otherwise, the nonparametric Kruskal–Wallis with Dunn’s *post-hoc* test was used. Additionally, the categorical variables in this study are shown as count and percentage, and these data were analyzed using the χ^2^ test. For multiple linear regression analysis of the correlations between serum CCL17 concentrations and baPWV, the analysis was performed with adjusted for age sex, hypertension, BMI, LDL-C, smoking status, and diabetes mellitus. The software GraphPad Prism 9 was applied for statistical analysis.

**References**

1 Zhang, Y. *et al.* CCL17 acts as a novel therapeutic target in pathological cardiac hypertrophy and heart failure. *J Exp Med* **219**, e20200418 (2022).

2 Tang, X. *et al.* SIRT2 acts as a cardioprotective deacetylase in pathological cardiac hypertrophy. *Circulation* **136**, 2051-2067 (2017).

3 Wang, X. *et al.* Visualizing CD4 T-cell migration into inflamed skin and its inhibition by CCR4/CCR10 blockades using in vivo imaging model. *Br. J. Dermatol.* **162**, 487-496 (2010).

4 Wang, L. *et al.* CXCL1-CXCR2 axis mediates angiotensin II-induced cardiac hypertrophy and remodelling through regulation of monocyte infiltration. *Eur Heart J* **39**, 1818-1831 (2018).

5 Diaz-Canestro, C. *et al.* MMP-2 knockdown blunts age-dependent carotid stiffness by decreasing elastin degradation and augmenting eNOS activation. *Cardiovascular Research* **118**, 2385-2396 (2021).

6 Yang, X. F., Ding, F. M., Ye, Y. C. & Zhang, S. Y. Relationship between Neutrophil-to-Lymphocyte Ratio and Pulse Wave Velocity in Young Patients with Systemic Lupus Erythematosus. *Chin Med J (Engl)* **131**, 10-15 (2018).

7 Lu, Y. *et al.* Trajectories of Age-Related Arterial Stiffness in Chinese Men and Women. *Journal of the American College of Cardiology* **75**, 870-880 (2020).

8 Zheng, M. *et al.* Arterial Stiffness Preceding Diabetes. *Circ. Res.* **127**, 1491-1498 (2020).

9 Kan, H. *et al.* Single-cell transcriptome analysis reveals cellular heterogeneity in the ascending aortas of normal and high-fat diet-fed mice. *Exp. Mol. Med.* **53**, 1379-1389 (2021).


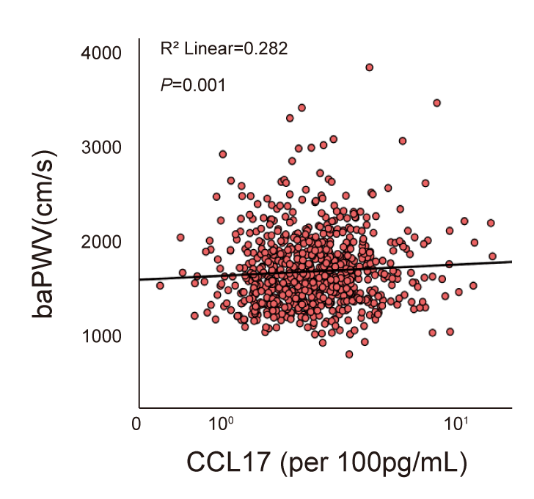


Figure. S1. Correlation between CCL17 and baPWV. The multiple linear regression analysis was performed to test correlation between CCL17 concentrations and baPWV. This analysis was adjusted by age, sex, BMI, smoking status, LDL-C, hypertension, and diabetes mellitus.


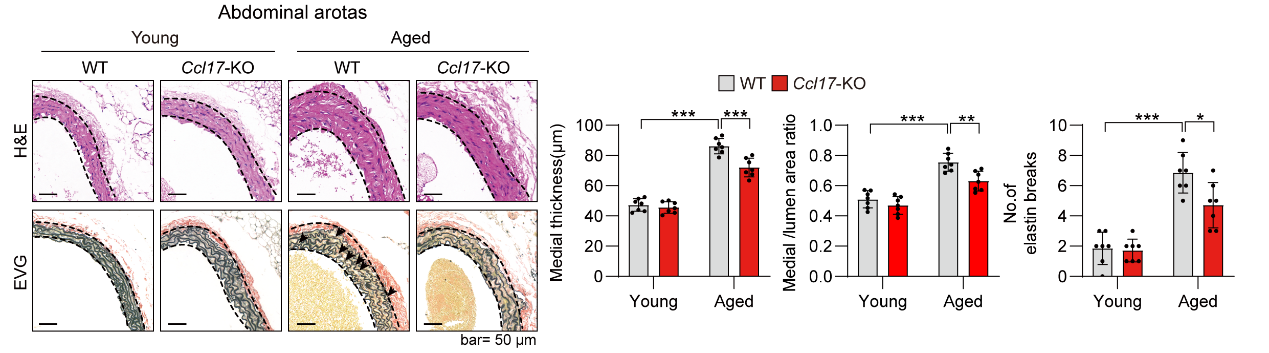


Figure. S2. *Ccl17* knockout represses aging-induced remodeling of abdominal aortas. H&E staining showing *Ccl17* knockout repressed aging-induced increase in thickness of abdominal aortas in mice, representative images and quantitative results of the medial thickness and the media-area/vessel-lumen ratio are shown (n=7). Elastic van Gieson (EVG) staining showing *Ccl17* knockout repressed aging-induced breakage of elastin fibers in abdominal aortas in mice, representative images and the number of elastin fiber breakages are shown (n=7). Arrows denote elastin breakage. All of the data are presented as mean ± SD. We used a two-way ANOVA with Bonferroni *post-hoc* test for multiple comparison when the assumptions were satisfied. Otherwise, the Kruskal–Wallis with Dunn’s *post-hoc* test was applied. **P*<0.05, ****P*<0.001.


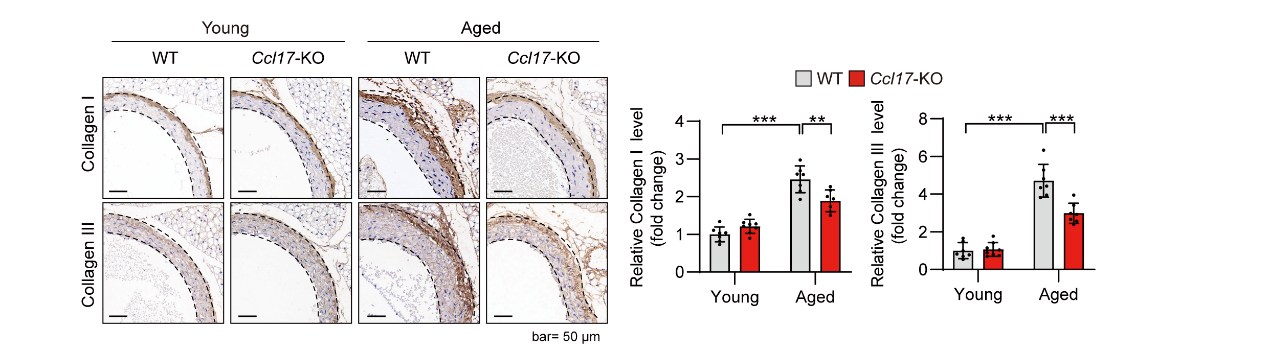


Figure. S3. *Ccl17* knockout represses aging-induced expression of collagens in the aortas. An immunohistochemical staining assay was performed to test Collagen I and III in the thoracic aorta in young and aged mice with/without *Ccl17* knockout (n=7-8). All the data are presented as mean ± SD. We used a two-way ANOVA for statistical analysis, with Bonferroni *post-hoc* test for multiple comparisons. ***P*<0.01, ****P*<0.001.


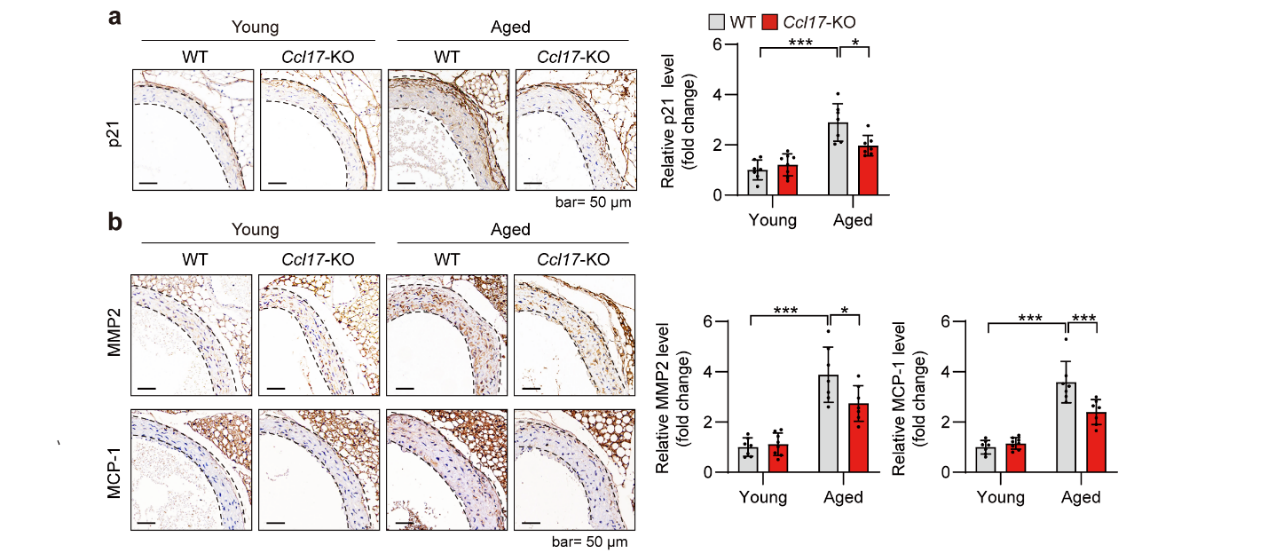
**Figure. S4. *Ccl17* knockout represses aging-induced senescence markers in the aortas.** (**a**) An immunohistochemical staining assay was performed to test the level of the senescence marker p21 in the thoracic aorta in young and aged mice with/without *Ccl17* knockout. Representative images and quantitative results of p21 levels are shown (n=7-8). (**b**) An immunohistochemical staining assay was performed to test the level of the biomarkers of senescence-associated secretory phenotypes (SASP) in thoracic aorta in young and aged mice with/without *Ccl17* knockout. Representative images and quantitative results of MMP2 and MCP-1 levels are shown (n=7-8). All the data are presented as mean ± SD. A two-way ANOVA with Bonferroni *post-hoc* test was applied, when the assumptions were satisfied to correct for multiple comparison. Otherwise, the nonparametric Kruskal–Wallis with Dunn’s *post hoc* test was performed for multiple comparison. **P*<0.05, ****P*<0.001.


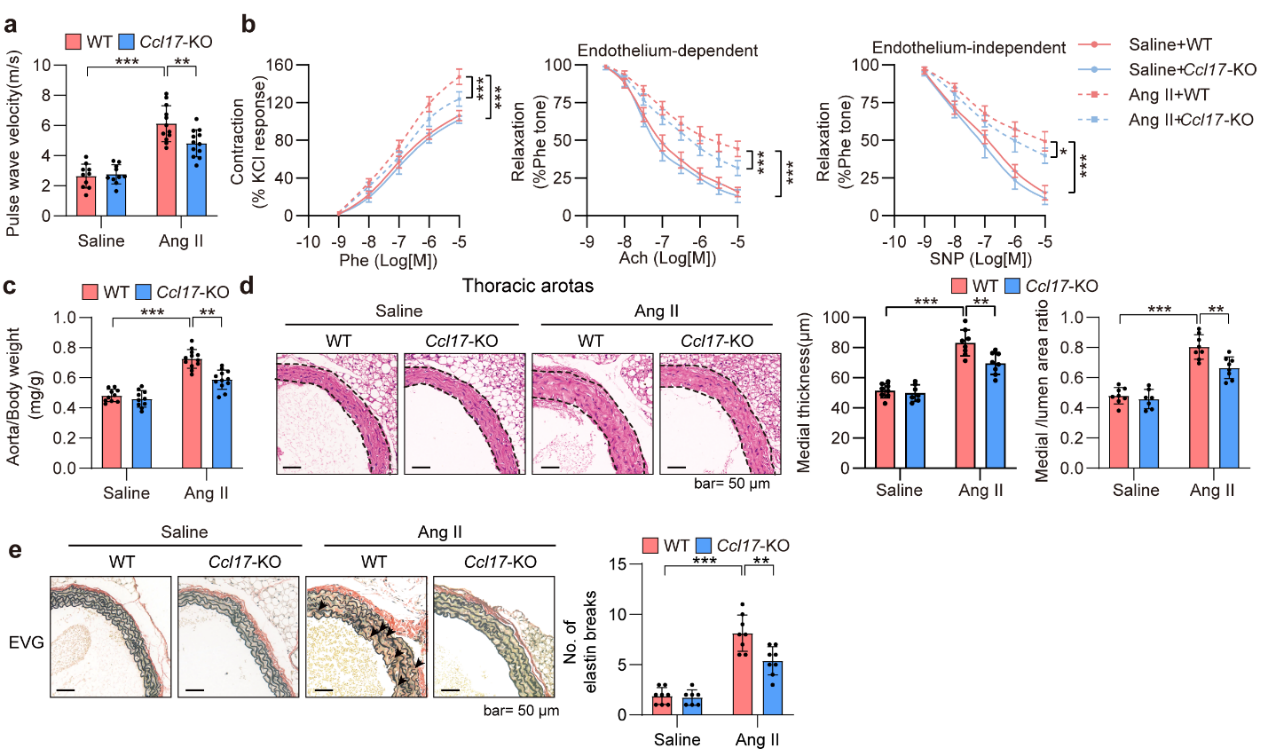
**Figure. S5. *Ccl17* deficiency represses dysfunction and remodeling of aortas induced by Ang II.** *Ccl17* knockout repressed angiotensin II (Ang II)-induced vascular dysfunction. Young (4-month) WT and *Ccl17*-KO male mice were challenged by Ang II (1.3 mg/kg per day) for four weeks, and then the aortas were analyzed. (**a**) PWV measurement revealed that *Ccl17* knockout repressed an Ang II-induced increase in arterial stiffness (n=10-12). (**b**) *Ex vivo* analysis of the vascular constriction-relaxation function of aortas. (left) arterial vessel contractions mediated through phenylephrine; (middle) endothelium-dependent relaxation responding to acetylcholine; (right) endothelium-independent relaxation responding to sodium nitroprusside (n=6). (**c**) *Ccl17* knockout reduced the aorta-weight/body-weight ratio in Ang II-challenged mice (n=10-12). (**d**) *Ccl17* knockout inhibited vascular remodeling in Ang II-challenged mice; H&E staining of the thoracic aortas was performed, and the medial thickness and the media-area/vessel-lumen ratio were quantified (n=7-8). (**e**) EVG staining showing *Ccl17* knockout repressed Ang II-induced breakage of elastin fibers in abdominal aortas in mice. Representative images and the number of elastin fiber breakages are shown (n=7-8). Arrows denote elastin breakage. All of the data are presented as mean ± SD. A two-way ANOVA with Bonferroni *post-hoc* test was applied, when the assumptions were satisfied, to correct for multiple comparison. Otherwise, the nonparametric Kruskal–Wallis test was performed, with Dunn’s *post-hoc* test for multiple comparison. ***P*<0.01, ****P*<0.001.


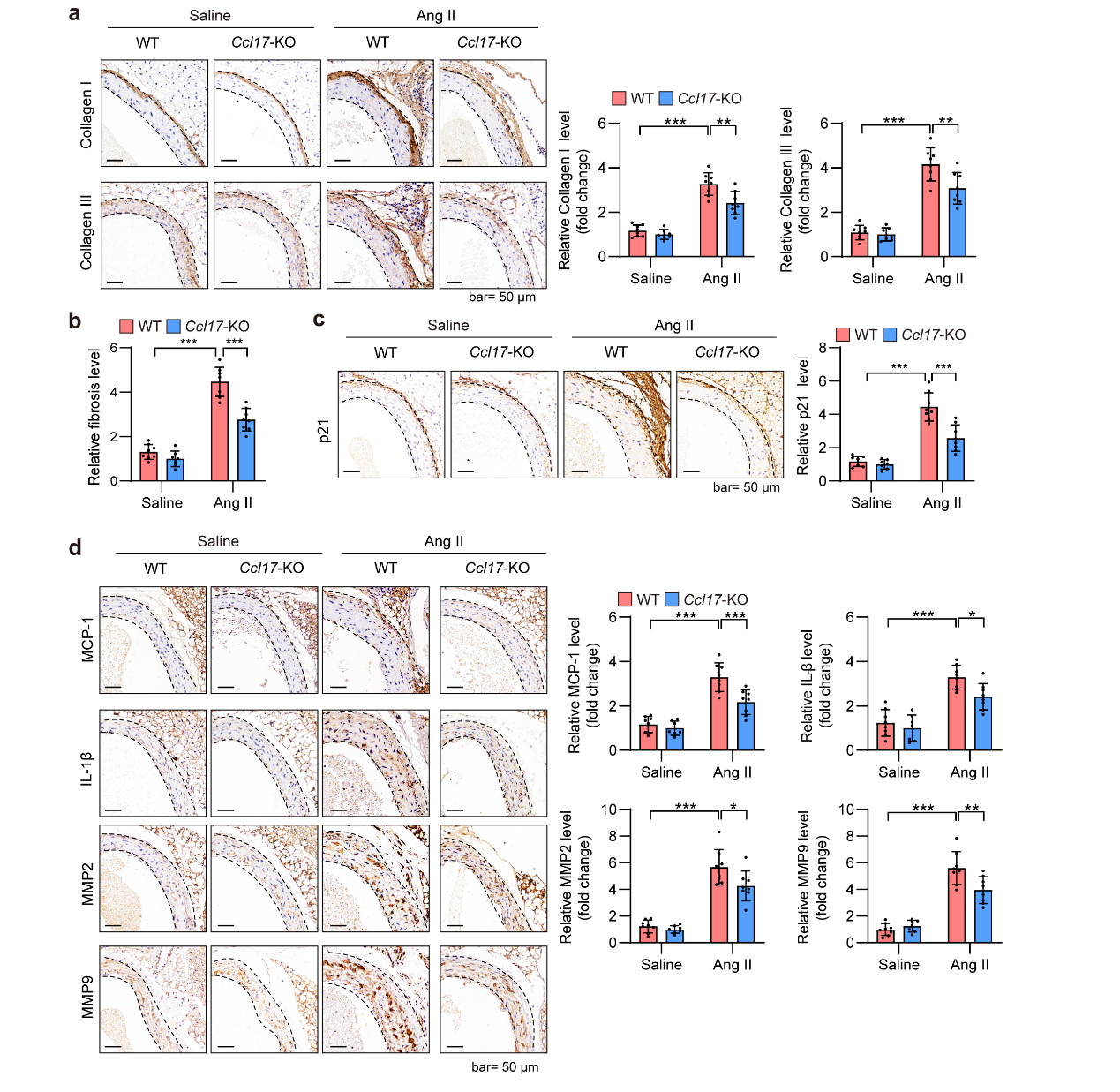


**Figure. S6. *Ccl17* knockout represses Ang II-induced fibrosis and senescence of the aortas.** (**a**) An immunohistochemical staining assay was used to test Collagen I and III in the thoracic aorta in young Ang II-treated mice with/without *Ccl17* knockout (n=7-8). (**b**) Quantitative results of fibrosis level are shown (n=7-8). The analysis was based on Masson staining. (**c**) An immunohistochemical staining assay was performed to test the senescence marker p21 in the thoracic aorta in young Ang II-treated mice with/without *Ccl17* knockout. Representative images and quantitative results of p21 levels are shown (n=7-8). (**d**) An immunohistochemical staining assay was performed to test the biomarkers of senescence-associated secretory phenotypes (SASP) in thoracic aorta in young Ang II-treated mice with/without *Ccl17* knockout (n=7-8). All the data are presented as mean ± SD. When the assumptions were satisfied, a two-way ANOVA with Bonferroni *post-hoc* test was applied for multiple comparison. Otherwise, the nonparametric Kruskal–Wallis test was used, with Dunn’s *post hoc* test for multiple comparison. **P*<0.05, ***P*<0.01, ****P*<0.001.


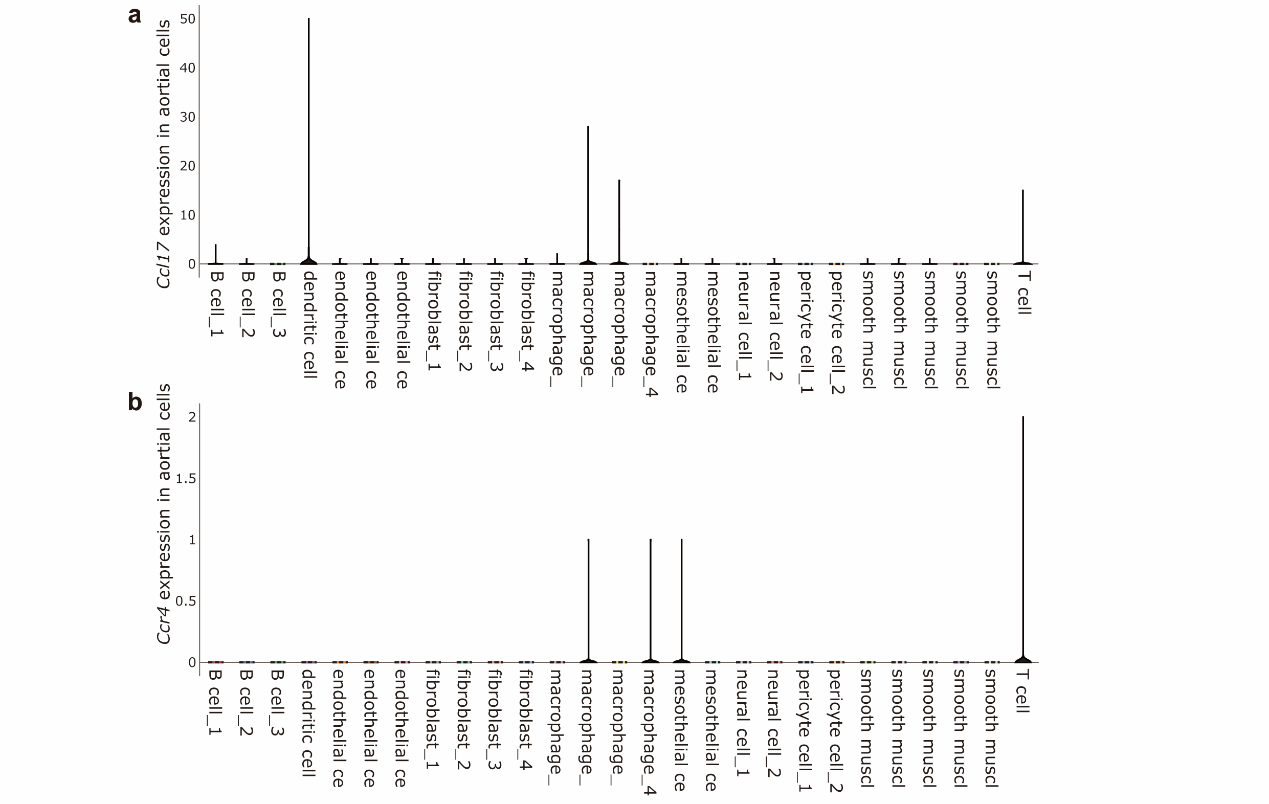


**Figure. S7. CCL17 and its receptor CCR4 in mouse aortas.** *Ccl17* and *Ccr4* mRNA in mouse aortas were analyzed using the web tool singlecell.broadinstitute.org. The dataset SCP1361 was used as input. (**a**) *Ccl17* mRNA level in aortic cells. (**b**) *Ccr4* mRNA levels in aortic cells.


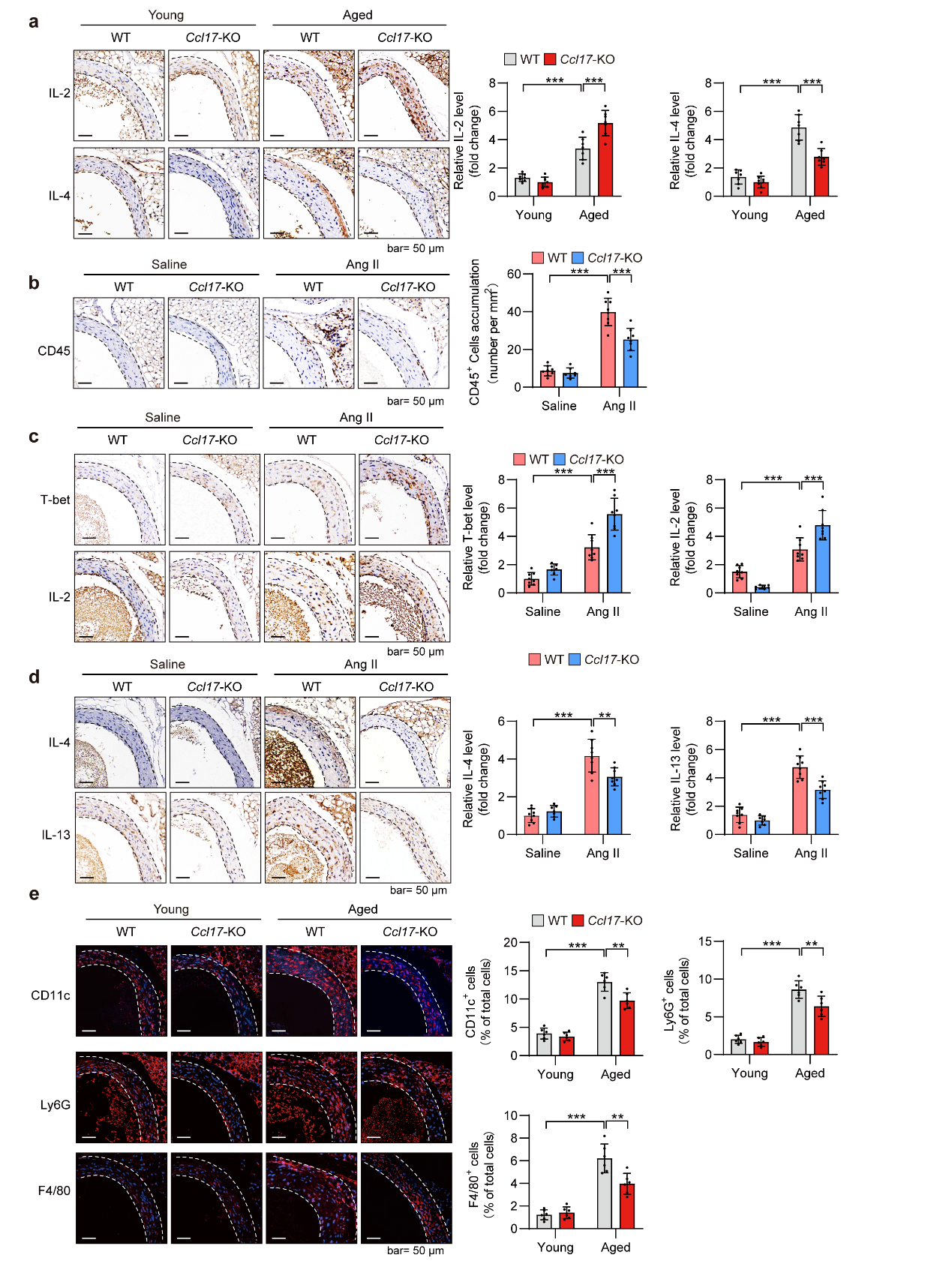


**Figure. S8. *Ccl17* regulates T-cell polarization and the immune microenvironment in the aortas.** (**a**) *Ccl17* knockout increased the expression of Th1 marker IL-2 and reduces Th2 marker IL-4 in aged aortas (n=7-8). (**b**) *Ccl17* knockout reduced immune cells in Ang II-infused aortas. Representative images and quantitative results are shown (n=7-8). (**c**) *Ccl17* knockout upregulated Th1 markers T-bet and IL-2 in the aortas from Ang II-challenged mice (n=7-8). (**d**) *Ccl17* knockout reduced Th2 markers IL-4 and IL-13 in the aortas from Ang II-challenged mice (n=7-8). (**e**) *Ccl17* knockout reduced infiltration of dendric cells (CD11c^+^), neutrophils (Ly6G^+^), and macrophages (F4/80^+^) in aged aortas (n=7-8). All the values are shown as mean ± SD. When the assumptions were satisfied, a two-way ANOVA test with Bonferroni *post-hoc* test was applied. Otherwise, the nonparametric Kruskal–Wallis test with Dunn’s *post hoc* test was used. ***P*<0.01, ****P*<0.001.


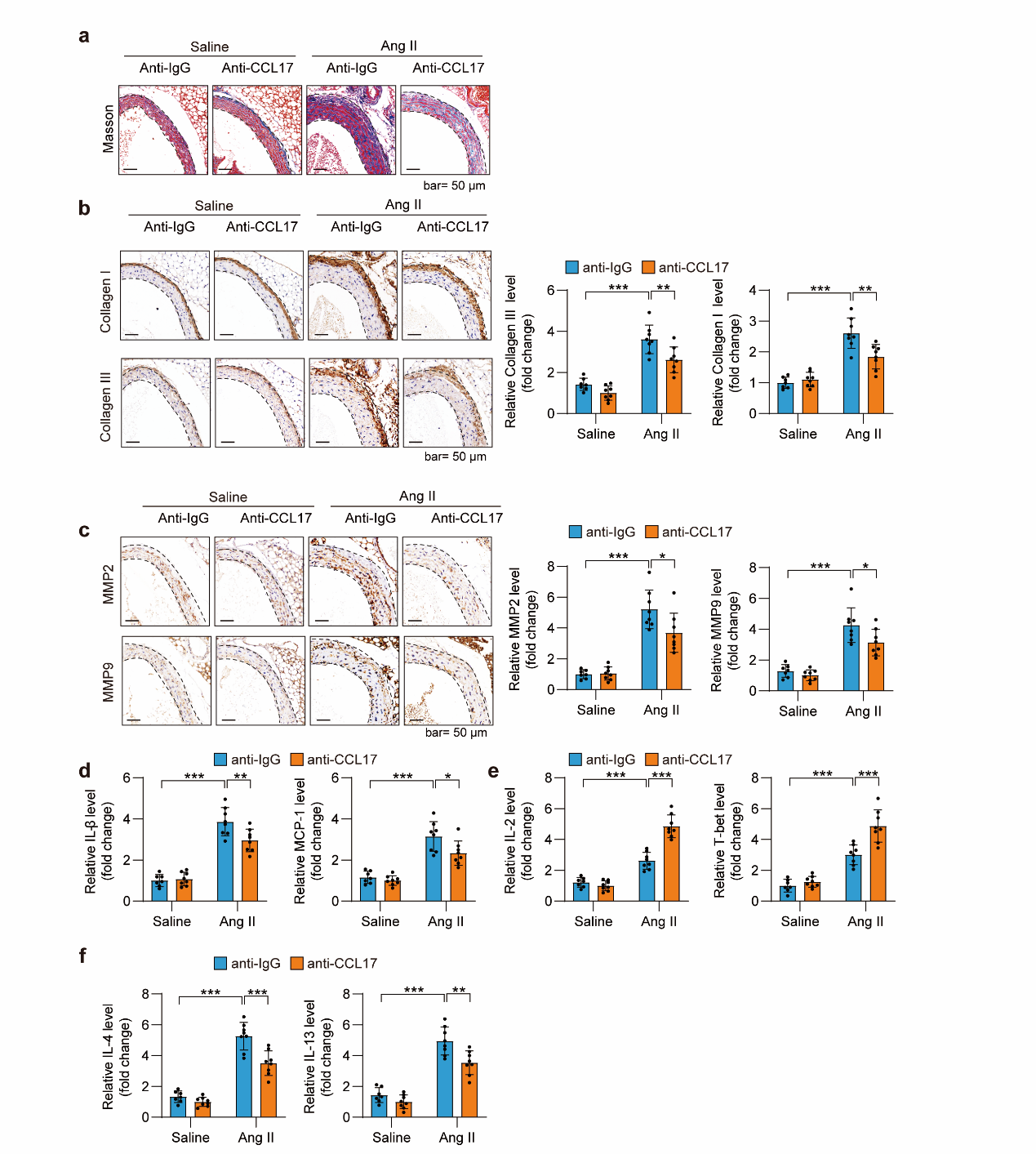


**Figure. S9. CCL17 antibody represses Ang II-induced vascular fibrosis, SASP, and T cell polarization**. (**a**) Masson staining showing CCL17 neutralizing antibody inhibited vascular fibrosis. Quantitative results are shown in the main figure. (**b**) Immunohistochemical staining of Collagen I and III showing CCL17 neutralizing antibody inhibited vascular fibrosis. Representative images and quantitative results are shown (n=7-8). (**c**) Immunohistochemical staining of SASP biomarkers MMP2 and MMP9. Representative images and quantitative results are shown (n=7-8). (**d**) CCL17 neutralizing antibody inhibited SASP biomarkers IL-1β and MCP-1 (n=7-8). (**e**) CCL17 neutralizing antibody upregulated Th1 markers T-bet and IL-2 (n=7-8). (**f**) CCL17 neutralizing antibody reduced Th2 markers IL-4 and IL-13 (n=7-8). All the values are shown as mean ± SD. When the assumptions were satisfied, a two-way ANOVA with Bonferroni *post-hoc* test was applied for multiple comparison. Otherwise, the nonparametric Kruskal–Wallis with Dunn’s *post hoc* test was performed. **P*<0.05, ***P*<0.01, ****P*<0.001.

**Table S1. Basic clinical information of human populations.**

|  | **Young population（age<60 yr; n=570)** | **Old population (age ≥60 yr; n=471)** | ***P* value** |
| --- | --- | --- | --- |
| Age (y) | 50.24±5.94 | 67.96±7.48 | <0.001 |
| Gender (male %) | 221(38.77) | 203(43.10) | 0.164 |
| Height(cm) | 160.06±7.57 | 156.74±7.77 | <0.001 |
| Weight(kg) | 68.5±11.48 | 64.40±11.24 | <0.001 |
| Body mass index (kg/m^2^) | 26.68±3.77 | 26.17±3.87 | 0.031 |
| Systolic blood pressure (mmHg) | 127.87±17.24 | 138.74±20.48 | <0.001 |
|  |  |  |  |
| Diastolic blood pressure (mmHg) | 79.22±10.9 | 78.05±10.63 | 0.082 |
| Smoking (Current smoker %) | 170(29.82) | 165(35.03) | 0.083 |
| Total cholesterol (mmol/L) | 4.52±0.92 | 4.82±0.97 | <0.001 |
| Triglyceride (mmol/L) | 1.53±1.46 | 1.57±1.15 | 0.618 |
| High-density lipoprotein cholesterol (mmol/L) | 1.24±0.32 | 1.26±0.30 | 0.266 |
| Low-density lipoprotein cholesterol (mmol/L) | 2.62±0.75 | 2.87±0.84 | <0.001 |
| Diabetes mellitus | 67(11.75) | 106(22.5) | <0.001 |
| Glucose(mmol/L) | 5.88±1.82 | 6.25±1.93 | 0.001 |
| CCL17 (pg/mL) | 239.05±130.70 | 289.30±246.11 | <0.001 |
| baPWV (cm/s) | 1437.59±221.76 | 1745.00±336.86 | <0.001 |

All the values are shown as means ± SD or n (%).

**Table S2. Serum CCL17 level in mice treated with/ without anti-CCL17 antibody.**

|  | **Saline (n=8)** | **Ang II+anti-IgG (n=8)** | **Ang II+anti-CCL17 (n=8)** |
| --- | --- | --- | --- |
| CCL17 (pg/ml) | 335.0±62.54 | 538.1±107.2*** | 355.5±54.09^##^ |

The experiments were performed as we described in our recent paper J Exp Med. 2022; 219: e20200418. This basal information was also shown in that previous paper Figure 5B. All the data are presented as mean ± SD. A two-way ANOVA with Bonferroni *post-hoc* test was applied. ****P*<0.001 *vs*. Saline, ^##^*P*<0.01 *vs.* Ang II+anti-IgG.

**Table S3. Heart rate and blood pressure of mice treated with/ without anti-CCL17 antibody.**

|  | **Saline+anti-IgG (n=12)** | **Saline+anti-CCL17 (n=12)** | **Ang II +anti-IgG (n=13)** | **Ang II+anti-CCL17 (n=13)** |
| --- | --- | --- | --- | --- |
| Heart rate (bpm) | 601.6±53.42 | 604.7±30.26 | 600.8±26.98 | 597.2±32.67 |
| DBP (mmHg) | 63.83±6.820 | 61.08±10.01 | 96.85±8.707*** | 93.85±8.562 |
| SBP (mmHg) | 104.0±11.79 | 99.83±8.077 | 145.4±11.14*** | 142.5±11.21 |

The experiments were performed as we described in our recent paper J Exp Med. 2022; 219: e20200418. This basal information was also shown in that previous paper Figure S5A-B. All the values are shown as mean ± SD. A two-way ANOVA test with Bonferroni *post-hoc* test was used. ****P*<0.001 *vs*. Saline+anti-IgG.
